# Key Components for the Delivery of Palliative and End-of-Life Care in Care Homes in Hong Kong: A Modified Delphi Study

**DOI:** 10.3390/ijerph19020667

**Published:** 2022-01-07

**Authors:** Helen Yue-Lai Chan, Cecilia Nim-Chee Chan, Chui-Wah Man, Alice Dik-Wah Chiu, Faith Chun-Fong Liu, Edward Man-Fuk Leung

**Affiliations:** 1 The Nethersole School of Nursing, The Chinese University of Hong Kong, Hong Kong SAR, China; 2 The Hong Kong Association of Gerontology, Hong Kong SAR, China; channcc@hkag.org (C.N.-C.C.); mancw@hotmail.com (C.-W.M.); alicechiu@hkag.org (A.D.-W.C.); faithliu@hkag.org (F.C.-F.L.); emfleung@yahoo.com.hk (E.M.-F.L.)

**Keywords:** palliative care, end of life, long-term care, older adults, residential care home, quality of care, service development

## Abstract

Integrating the palliative care approach into care home service to address the complex care needs of older adults with frailty or advanced diseases has been increasingly recognized. However, such a service is underdeveloped in Hong Kong owing to socio-cultural and legal concerns. We adopted a modified Delphi study design to identify the key components for the delivery of palliative and end-of-life care in care home settings for the local context. It was an iterative staged method to assimilate views of experts in aged care, palliative care, and care home management. A multidisciplinary expert panel of 18 members consented to participate in the study. They rated their level of agreement with 61 candidate statements identified through a scoping review in two rounds of anonymous surveys. The steering group revised the statements in light of the survey findings. Eventually, the finalized list included 28 key statements concerning structure and process of care in seven domains, namely policy and infrastructure, education, assessment, symptom management, communication, care for dying patients, and family support. The findings of this study underscored concerns regarding the feasibility of statements devised at different levels of palliative care development. This list would be instrumental for regions where the development of palliative and end-of-life care services in care home setting is at an initial stage.

## 1. Introduction

The need for integrating the palliative care approach into care home services is gaining recognition [1,2,3,4,5,6]. Care homes, also known as nursing homes in some regions, often are the last place of care for older adults with frailty or advanced diseases, owing to the need for long-term professional care [7]. Hence, care home residents are at a higher risk of mortality than their home-dwelling counterparts, accounting for approximately 30% of all deaths [1,8].

Given the inherited differences in the nature and resources between care homes and hospitals, specific policies and guidelines are formulated to guide the development of palliative and end-of-life care in care home settings [9]. An evidence-based guideline “Guidelines for a Palliative Approach in Residential Aged Care” developed in Australia in 2004, was among the first of its kind [10]. It adopts a holistic approach to assess and address physio–psychosocial–spiritual needs of the residents and their family members. The Regulation and Quality Improvement Authority of the Northern Ireland (2013) also formulated a set of guidelines to provide specific guidance about illness identification, palliative care assessment, advance care planning and coordination of care, care for the dying, care after death, and bereavement and staff support and training [11].

Lately, the focus of these guidelines has expanded beyond an individual level of care. In addition to the physical, psychological, and social aspects of care, the palliative care practice guidelines and standards for nursing homes developed by Temkin-Greener and associates (2015) also cover the structure and processes of care, as well as the cultural, ethical, and legal aspects of care [12]. The framework developed recently by the European Association for Palliative Care for palliative care implementation in long-term care facilities also spans over organizational (micro), inter-sectoral (meso), and system (macro) levels [13]. These trends of development suggest that the influence of external factors should also be taken into consideration in service delivery. 

Unlike European countries [6], the development of palliative and end-of-life care services in care home setting in Hong Kong is in its infancy stage. A care home is not a legally recognized place of death [14]. A local public opinion survey also showed that only a small proportion (16.2%–34.7%) of respondents preferred care homes as a place of death [15,16]. Residents whose health conditions have deteriorated are generally sent to hospitals for medical care due to concerns of legal liability and inadequate medical expertise and facilities in care homes [17,18] Therefore, the existing guidelines and framework developed in other regions might have limited applicability to Hong Kong.

## 2. Materials and Methods

**Figure 1:**
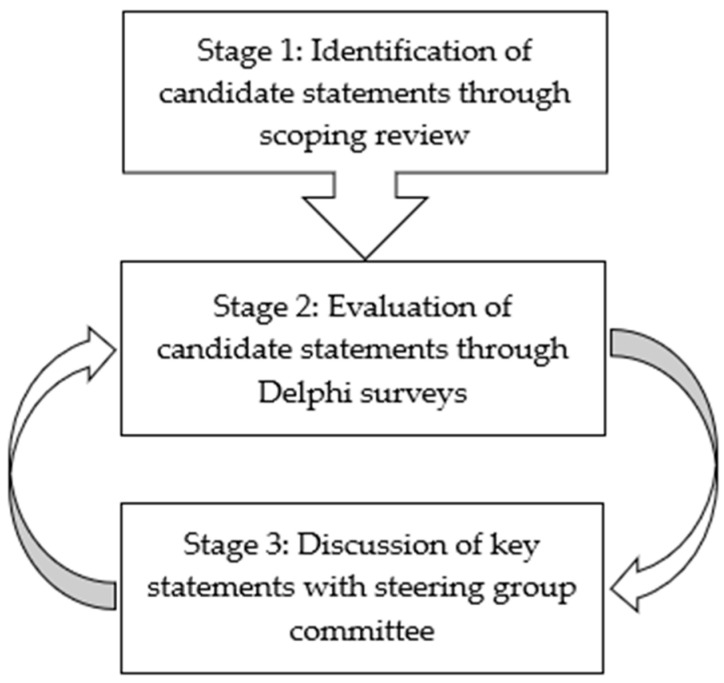
Flow Diagram of the Study.

This study aims to identify the key components for the delivery of palliative and end-of-life care in care home setting in Hong Kong. We adopted the modified Delphi method to define the components for the delivery of palliative and end-of-life care in care homes on the basis of expert consensus. The Delphi method is an effective strategy to assimilate expert opinions into group consensus for a specialized area, including palliative care [19,20]. The iterative multistage consultative process enables consensus building among experts of various expertise related to the field (Figure 1).

### 2.1. Stage 1: Identification of Candidate Statements 

A scoping review was conducted to identify a list of conditions related to the provision of palliative and end-of-life care in care home settings according to the Preferred Reporting Items for Systematic Reviews and meta-analyses (PRISMA) reporting guidelines extended for scoping review [21]. Scoping review is useful to examine the extent and nature of research activities, summarize the findings, and identify research gaps in a complex topic across a broad range of study designs. We initially searched for articles published in English between 2008 and 2017 using MEDLINE via Ovid, CINAHL via EBSCO, PsycINFO, and the Cochrane Library. The following keywords were used: (end of life OR terminal care OR palliative care) AND (care home* OR nursing home* OR care facility* OR residential OR long-term care) AND (quality indicator OR quality criteria OR quality of care OR quality improvement OR quality management). The inclusion criteria were all types of studies related to palliative or end-of-life care in care home setting that were published after 2010, so as to ensure applicability to the current context. We further searched for practice guidelines, reports, and policy documents issued by government or professional bodies using internet search engines. The reference lists of the retrieved documents and articles were reviewed to hand search for potential papers. Editorials, discussion papers, conference abstracts, study protocols, and articles that only reported residents’ preferences for end-of-life care and place of death were excluded. Three core group members (C.M., A.C., and F.L.) reviewed the documents and extracted statements relevant to the service delivery. 

### 2.2. Stage 2: Evaluation of Candidate Statements through Delphi Surveys 

Two rounds of the Delphi survey were conducted with an expert panel with multidisciplinary expertise in geriatrics, palliative, and care home services. Panelists were identified through networks of the steering group and a professional society in aged care. There was no consensus on the number of panelists to be included in the Delphi study, with the panel sizes ranging widely from 15 to 60 [19,22]. Instead, the qualities and diversity of the panelists are considered as more important than the number [23]. To ensure the quality of the panelists, purposive sampling was employed to ensure representatives of different specialties, disciplines, and roles were included. An information sheet explaining the study nature and purpose and a questionnaire listing all the candidate statements identified in stage 1 were distributed directly through post or email to the potential panelists. Those who consented to participate were asked to rate their level of agreement with the candidate statements independently using a five-point Likert scale, from 1 = strongly disagree to 5 = strongly agree. They were also welcomed to provide free-text comments in the questionnaire. The statements were then revised according to the results of the first round of survey and were sent to the panel again for another round of survey. All participation was on a voluntary basis and the returned questionnaires were anonymous to ensure genuine responses. 

### 2.3. Stage 3: Discussion of Key Statements 

Between the surveys, the project team (all authors) and the steering group committee had meetings to discuss and revise the statements and framework on the basis of the survey findings. The rating for each statement was presented using median, with a higher median indicating a higher level of agreement, and sum of percentages of agree and strongly agree. The qualitative comments were also collated for discussion. 

## 3. Results

### 3.1. Identification of Candidate Statements 

In the scoping review, the search yielded 1165 potentially relevant documents. After de-duplication, the titles and abstracts of 255 documents were screened to determine eligibility. Then, 206 document that did not meet the selection criteria were removed after initial screening. The remaining 49 articles were included for full-text review and another seven articles were subsequently excluded due to ineligibility. Another 11 documents identified through hand search were added. Therefore, 53 documents of different nature, including prospective and retrospective interventional studies, case studies, cross-sectional surveys, and qualitative studies and guidelines, were included for full-text review. From which, a total of 61 candidate statements were identified and categorized into 13 domains, namely, policy, philosophy of care, organizational support, environment and facilities, staffing and training, identification of care needs, physical care, pain and symptom management, psychosocial care, spiritual care, coordination and collaboration, communication, and family support.

### 3.2. Results of Delphi Surveys

Out of the 50 experts approached, 18 consented to participate and complete the survey, giving a response rate of 36% in round 1 and 100% in round 2. They included 4 physicians, 10 nurses, 2 social workers, and 2 allied health professionals. Their mean age was 50.8 (SD 12.0) years, with an average of 21.9 (SD 13.0) years of clinical experience in aged care or palliative care.

Table 1 shows the median and percentage of agreement for each statement in the round 1 survey. The median rating of the statements was generally at 4 or above, except for two statements, namely “Including trained volunteers in care delivery” and “Exploring the spiritual, religious, and existential concerns of residents and family members”. The percentage of agreement of these two statements were 27.8% and 50.0%, respectively. Statements with the highest median were related to staff education and support, symptom management, and information giving. The qualitative comments suggested that some statements were general care principles and should always be upheld, regardless of the residents’ health conditions. Examples are “promoting person-centered and holistic care” and “maintaining residents’ dignity, privacy, and autonomy” related to the philosophy of care and “providing basic care to maintain cleanliness and comfort,” in the physical care domain. These statements were removed because they were not specific to palliative and end-of-life care. 

Some statements concerning similar issues were merged. For example, several statements related to staff training and coaching in palliative and end-of-life care were combined into one statement. Some wordings were rephrased or edited to clarify the meaning. Domains concerning similar issues were assimilated; for instance, policy, organizational support, and physical environment were integrated into a single domain of policy and infrastructure. Some wordings were also revised to enhance clarity. On the other hand, five statements that were considered as unique and important for palliative and end-of-life care in care home setting were newly added, such as “Including nutritional screening in the initial assessment”, “Formulate and regularly review nutrition and hydration care plan” and “Managing the prescription of controlled drugs according to the local laws”.

The finalized list includes 28 statements categorized into seven domains, as shown in Table 2. In the subsequent round of survey, the median scores for all statements were at 4.0 or above.

## 4. Discussion

### 4.1. Summary of Key Findings

This study is the first of its kind to identify the key components for delivering palliative and end-of-life care in care home setting in Chinese communities. Through the modified Delphi method, a finalized list of 28 statements covering seven domains, namely, policy and infrastructure, education, assessment, symptom management, communication, care for dying patients, and family support, was generated. This set of statements lays down the core elements required for palliative and end-of-life care service development in care home settings in Hong Kong.

### 4.2. Conceptual Framework

We adopted the Donabedian model for mapping the statements because this model has been widely used for evaluating the quality of health services [24]. The structure of the care domain covers the external factors, such as policies, environment, capacities, and resources, for supporting the care service development, whereas the process of the care domain focuses on the activities and interactions required for care delivery [24]. In the finalized list of statements in this study, 5 were in the structure of care dimension and 23 were in the process of care dimension. The structure of care domain clarifies the elements that should be in place at an organizational level to facilitate the development and implementation of palliative and end-of-life care in a care home setting [25]. The process of the care domain provides guidance on the purposes and crucial elements of care [18,26].

### 4.3. Tailored to the Socio-Cultural Context

Delphi study is commonly used in health care to achieve an international expert consensus for developing clinical guidelines or for defining newly emerged concepts [19,22,27]. However, the findings of this present study suggest that the appropriateness and feasibility of the statements were major concerns of the panelists, thereby highlighting the importance of deriving statements that are socio-culturally specific to the local context. While the findings suggested that most of the panelists agreed in principle with the statements identified from the literature, their comments suggested that they paid more attention to the practicality of the statements derived. In Hong Kong, care homes generally are not regarded as a place of death, and thus, in addition to concern about the infrastructure, much attention was placed on concern of liability, such as prescription of anticipatory medication, involvement of volunteers in the care provision, and supporting dying in place, under the existing constraints of limited professional support and manpower shortage in care home setting. Moreover, concerns regarding the competence of care home staff for the delivery of palliative and end-of-life care was underscored. This echoes previous studies, that staff education is a cornerstone for the provision of quality palliative and end-of-life care [18,28,29,30]. In the Delphi study conducted by Temkin-Greener and associates, discrepancies between the ratings of importance and feasibility regarding the palliative care practice guidelines in care homes were also noted, because some of the expectations were out of the care home control [12]. This observation highlights the importance of devising specific guidance for the local context.

### 4.4. Strengths 

The strength of this study is that a systematic staged approach was adopted to ensure the specificity and applicability of the findings. The candidate statements were synthesized from a scoping review to ensure a breadth of components in the recent development were taken into account. The expert panel encompassed experienced representatives from different disciplines and specialties. The study results might also be applicable to neighborhood cities in the mainland China with similar cultural context and levels of palliative care development.

### 4.5. Limitations

We acknowledge several limitations of this study. First, this study focused on the Hong Kong context, where the delivery of palliative and end-of-life in care home setting is a new initiative. Its generalizability to other communities with different levels of development in aged care and palliative care cannot be ascertained yet. Second, the response rate for the Delphi survey is relatively low and could not achieve Sumsion’s standard (i.e., 70%) [31], although it is comparable with the median number of panelists reported in a systematic review of the Delphi study [22]. One possible reason for the low response rate was the relatively lower levels of awareness, interest, and knowledge regarding palliative and end-of-life care when compared with other aspects of service development in care home setting, such as frailty prevention, dementia care, or infection control. A response bias cannot be precluded as those who participated in the surveys might be more supportive to the development of palliative and end-of-life care in care homes. Additionally, the panelists’ ratings were subjective due to the study nature.

## 5. Conclusions

The development of palliative and end-of-life care in care home setting in Hong Kong is in its infancy stage. Our study marks the first step in identifying key elements for service delivery. A staged systematic approach was adopted to incorporate views from multidisciplinary experts. This list of statements would be instrumental for providing guidance to relevant service development, and thereby benchmarking the quality of care. Further work is needed to evaluate the attainment of each statement within care homes along the service development process.

## Figures and Tables

**Table 1:** Findings of the round 1 survey.

Domain/Statements	Percentage of Agreement ^1^	Median ^2^
*I. Policy*		
1. Formulating a framework to promote end-of-life care practices in care home setting.	83.3	4.0
2. Developing an administrative policy specific to end-of-life care.	88.9	4.0
3. Developing clinical policies and protocols to encourage documentation of the care practices.	77.8	4.0
4. Having a professional body to provide guidance on the care implementation.	94.4	4.0
5. Having an external reviewer to visit and monitor how the care practices are provided in place.	72.2	4.0
*II. Philosophy of care*		
6. Adopting a shared care approach.	77.8	4.0
7. Treat residents with respect, kindness and dignity.	88.9	4.0
8. Promote person-centred, personalized, and holistic care.	88.9	4.0
9. Being responsive to the needs of patients and family members in a timely manner.	88.9	4.0
10. Preserving residents’ dignity, privacy, and autonomy.	88.9	4.0
*III. Organization support*		
11. Institutional leadership to support end-of-life care practices.	77.8	4.0
12. Building a quality assurance mechanism to monitor the quality of end-of-life care.	88.9	4.0
13. Reflecting on the quality of care in a continuous fashion.	77.8	4.0
14. Designing remembrance activities to encourage open atmosphere to talk about death.	83.3	4.0
15. Acknowledging the time devoted to support end-of-life care practices.	77.8	4.0
*IV. Staffing and training*		
16. Ensuring staff adequacy and staff–resident ratio for supporting end-of-life care.	66.7	4.0
17. Building a multidisciplinary team to address the complex care needs.	77.8	4.0
18. Providing specific staff training tailored to the context and needs of care home setting.	88.9	4.0
19. Including medico–ethical–legal issues in the training.	88.9	4.0
20. Providing mentoring to ancillary staff members.	77.8	4.0
21. Offering guidance at the bedside care.	88.9	5.0
22. Provide continuous staff education on a specific topic or issue.	88.9	5.0
23. Providing support to staff through counselling, debriefing, and bereavement care.	88.9	4.0
24. Foster a learning culture for sharing knowledge and information about clients’ conditions or needs.	77.8	4.0
*V. Environment and facilities*		
25. Providing a calm and private environment.	77.8	4.0
26. Creating a welcoming atmosphere to encourage family visitation.	88.9	4.0
27. Providing space and facilities for family members to stay overnight to accompany the resident in the last days of life.	66.7	4.0
*VI. Identification of care needs*		
28. Identifying the phase of illness of residents.	77.8	4.0
29. Conducting a structured and systematic approach for assessing physical, emotional, social, and spiritual needs of residents.	77.8	4.0
30. Timely detection of changes in residents’ health status.	77.8	4.0
31. Using valid and appropriate tools to assess the physical symptoms of the residents, especially for residents with cognitive impairment.	77.8	4.0
32. Assessing non-verbal cues of pain and distress (agitation and restless).	66.7	4.0
33. Formulate a supportive care plan for each resident to summarize the actual and anticipated needs.	55.6	4.0
*VII. Physical care*		
34. Ensuring adequate basic care to maintain cleanliness and comfort.	100.0	5.0
*VIII. Pain and symptom management*		
35. Adopting pharmacological and non-pharmacological strategies for symptom alleviation or control.	100.0	4.0
36. Regular medication review to manage symptoms.	88.9	4.0
37. Arrange prescription of anticipatory medication for possible distress.	72.2	4.0
38. Ongoing symptom management, in particular difficulties in breathing and nausea.	88.9	5.0
*IX. Psychosocial care*		
39. Providing counselling or reassurance to cope with stress and anxiety.	88.9	4.0
40. Including trained volunteers in care delivery.	27.8	3.0
41. Providing a supportive presence with the dying resident.	77.8	4.0
42. Providing bereavement care to family members.	77.8	4.0
*X. Spiritual care*		
43. Exploring the spiritual, religious, and existential concerns of residents and family members.	50.0	3.0
44. Providing support to the search for meaning, forgiveness, and reconciliation.	66.7	4.0
45. Providing or facilitating individual devotional activities for dying residents.	66.7	4.0
*XI. Coordination and collaboration*		
46. Designating a staff member as a named lead for the resident.	66.7	4.0
47. Holding regular meetings to discuss the residents’ care.	66.7	4.0
48. Maintaining a good rapport with the hospital, discharge planning coordinator, and community care team.	88.9	4.0
49. Maintaining communication about care, in particular out-of-service hours.	88.9	4.0
50. Collaborating with specialist palliative care team in complex cases.	88.9	4.0
51. Collaborating with general practitioners.	88.9	4.0
*XII. Communication*		
52. Preparing resident and family for end-of-life care (emotionally/intellectually).	77.8	4.0
53. Providing information to resident and family about end-of-life care and the dying process.	77.8	5.0
54. Initiating advance care planning with residents and family members to clarify preferred place of care and treatment preferences.	77.8	4.0
55. Planning for funeral arrangement.	55.6	4.0
56. Documenting the conversations within easy access.	55.6	4.0
57. Review the care plan regularly or when necessary.	88.9	4.0
*XIII. Family support*		
58. Promoting family involvement in care and decision-making.	88.9	4.0
59. Meeting with the family members on regular basis to explore their views towards the residents’ care.	88.9	4.0
60. Allowing the family and individuals of residents’ choice to be present in the dying phase.	88.9	4.0
61. Providing support to aftermath care and bereavement care to family members.	88.6	4.0

^1^ Percentages of agree and strongly agree; ^2^ range from 1 (strongly disagree) to 5 (strongly agree).

**Table 2:** Finalized list of statements.

Domains/Statements	Type ^1^
*A. Policy and infrastructure*	
1. Having policies and guidelines regarding palliative and end-of-life care.	S
2. Providing facilities to support the implementation of palliative and end-of-life care.	S
3. Providing private space for the family members and friends of the dying resident.	S
*B. Education*	
4. Providing training and supervision in palliative and end-of-life care to meet the staff’s learning needs.	S
5. Providing education for the residents and their family members to enhance their knowledge and understanding of palliative and end-of-life care.	S
*C. Assessment*	
6. Regularly assessing the residents’ care needs and documenting the results.	P
7. Using standardized assessment tools to periodically evaluate the pain experienced by the residents and the effects of pain management.	P
8. Including nutritional screening in the initial assessment.	P
9. Identifying the risks of poor nutrition, dehydration, or swallowing difficulties.	P
10. Formulating and regularly reviewing nutrition and hydration care plans.	P
*D. Symptom management*	
11. Managing physical symptoms and medication side effects in a systematic approach.	P
12. Managing the prescription of controlled drugs according to the local laws.	P
13. Providing adequate analgesics and/or sedatives for a dying resident to alleviate pain.	P
14. Making referrals to specialist palliative care services when deemed necessary due to the residents’ care needs.	P
*E. Communication*	
15. Regularly assessing residents, their family members, or representatives of the assessment results, based on their choices.	P
16. Recording and reviewing periodically end-of-life care preferences of the residents and their family members.	P
17. Including residents’ preferences and religious, spiritual, and cultural beliefs and involving their family members in advance care planning.	P
18. Consulting family members if the residents can no longer make decisions due to declining health.	P
19. Immediately notifying family members and other residents when a resident is detected with signs of impending death.	P
20. Providing emotional support for other residents and staff following a resident’s death and prepare a remembrance event based on the residents’ preferences.	P
*F. Care for dying residents*	
21. Providing care in a respectful manner with consideration of the cultural and religious practices of the dying resident.	P
22. Handling the deceased resident’s body according to guidelines, local laws, and regulations.	P
23. Making arrangements for the personal possessions of the deceased resident in a timely and respectful manner according to his/her preferences.	P
*G. Family support*	
24. Allowing sufficient time for the family members and friends to accompany the dying resident.	P
25. Providing family members with timely guidance or information regarding signs and symptoms of the dying resident.	P
26. Providing bereavement counseling for the family members or representatives.	P
27. Providing family members or representatives with information regarding registration of death and funeral arrangements.	P
28. Assessing the family needs for bereavement support services and providing information as necessary.	P

^1^ S, Structure of care; P, Process of care.

## Data Availability

The datasets used and/or analyzed during the current study are available from the corresponding author upon reasonable request.
